# Design and implementation of botulinum toxin on cricopharyngeal dysfunction guided by a combination of catheter balloon, ultrasound, and electromyography (BECURE) in patients with stroke: study protocol for a randomized, double-blinded, placebo-controlled trial

**DOI:** 10.1186/s13063-021-05195-8

**Published:** 2021-03-31

**Authors:** Mengshu Xie, Zulin Dou, Guifang Wan, Peishan Zeng, Hongmei Wen

**Affiliations:** Department of Rehabilitation Medicine, The Third Affiliated Hospital, Sun Yat-sen University, 600 Tianhe Road, Guangzhou, 510630 Guangdong Province China

**Keywords:** Botulinum toxin, Clinical trial, Cricopharyngeal achalasia, Cricopharyngeal dysfunction, Dysphagia, Protocol, Rehabilitation

## Abstract

**Background:**

Cricopharyngeal dysfunction (CPD) occurs in various neurological disorders, especially stroke. The treatment approaches of CPD include swallowing training, cricopharyngeal dilation, botulinum toxin (BTX) injection, and cricopharyngeal myotomy. BTX injection into the cricopharyngeal muscle is effective and safe for some patients with dysphagia, with a success rate between 43 and 100% (mean = 76%). However, well-designed randomized controlled clinical trials are needed to verify its efficacy and safety for the treatment of CPD. The objective of this study is to explore the efficacy and safety of BTX for neurogenic cricopharyngeal achalasia, when administering an injection into the cricopharyngeal muscle guided by a novel precise positioning method, that combines ultrasound, catheter balloon, and electromyography (BECURE).

**Methods:**

BECURE is a single-center randomized, placebo controlled, double-blinded, superiority clinical trial. To detect a significant difference between the 2 groups, a sample size of 44 patients is estimated. The intervention is BTX versus placebo, with 1:1 randomization. The randomization sequence from 1 to 44 was generated using the Statistical Package for Social Sciences. The study is divided into two phases. In the first phase, patients will be injected with BTX or the placebo. In the second phase, patients who received a placebo injection and those who did not respond to the first BTX injection will receive an injection of BTX. The primary outcome is the score of the Functional Oral Intake Scale (FOIS). The secondary outcomes are as follows: upper esophageal sphincter (UES) residual pressure, UES resting pressure, duration of UES relaxation, velopharyngeal and laryngopharyngeal peak pressure, UES opening, pharyngeal construction ratio, residue of bolus in the epiglottis valley or piriform sinus, and penetration and aspiration.

**Discussion:**

Dysphagia is a common complication of stroke. There is lack of high-quality evidence for the efficacy of BTX in treating neurogenic CPD. This study will clarify whether BTX injection into the cricopharyngeal muscle can be effective and safe for patients with stroke and CPD.

**Trial registration:**

Chinese Clinical Trial Register (ChiCTR1900025562). Registered on September 1, 2019.

## Background

Functional swallowing occurs due to a series of well-coordinated movements that allow transport of food bolus from the oral cavity into the stomach while avoiding passage of food bolus into the airway [1]. Dysphagia occurs due to neurological disorders that impair the transport of food from the oral cavity to the stomach efficiently and/or safely [2] and is associated with an increased risk of pulmonary complications, dehydration, malnutrition, and mortality [1, 3]. Dysphagia is a common complication in stroke patients with a morbidity rate between 20 and 90%, depending on the assessment methods [4].

The upper esophageal sphincter (UES) is the high-pressure physical barrier to the transport of a food bolus or liquids between the pharynx and esophagus. The cricopharyngeal muscle is main muscle responsible for UES function [5]. This muscle remains in a contracted position during respiration, preventing air from entering into the esophagus and relaxes and opens during swallowing or vomiting [2]. Cricopharyngeal dysfunction (CPD), characterized by difficulty of food bolus entering the esophagus, occurs often due to 3 main factors: cricopharyngeal muscle spasm, incomplete laryngeal movement, and insufficient descending pressure of the bolus [2].

Cricopharyngeal achalasia is one of the main manifestations of CPD characterized by an uncoordinated state of relaxation or spasm of the cricopharyngeal muscle. Primary cricopharyngeal achalasia is a rare, idiopathic disorder [6]. Secondary cricopharyngeal achalasia occurs in various disorders, including stroke, Parkinson’s disease, and head and neck cancers [7]. Cricopharyngeal achalasia is one of the main causes of dysphagia after stroke [8]. Many swallowing problems recover spontaneously within 7 days after stroke, but approximately 8 to 50% of patients experience persistent difficulties in swallowing 6 months after stroke [9, 10]. The prevalence rates of dysphagia vary due to differences in the definition of dysphagia, method of assessing swallowing function, timing of swallowing assessment after stroke, and number and type of patients with stroke studied [11]. Early therapy for dysphagia is effective and can reduce serious complications [12]. For patients with persistent dysphagia, more effective treatments are needed to reduce morbidity and mortality.

Several approaches exist for treating CPD, including swallowing rehabilitation, cricopharyngeal balloon dilation, botulinum toxin (BTX) injection, and cricopharyngeal myotomy [13]. Cricopharyngeal myotomy, a traditional surgical method, is the optimal choice for primary cricopharyngeal achalasia. However, its effectiveness for cricopharyngeal achalasia is considered unsatisfactory [14]. Furthermore, cricopharyngeal myotomy is unsuitable for patients in poor medical condition. Balloon dilation for neurogenic dysphagia has been widely used in clinical practice, and its usefulness has been supported [15]. However, we found that the swallowing function of some patients failed to improve even after prolonged balloon dilation. In one systematic review, the success rate of dilation was between 58 and 100% (mean = 81%) [13]. For patients with cricopharyngeal achalasia, BTX injection is useful [7, 16–18]. The success rates of BTX injection vary between 43 and 100% (mean = 76%) [13]. The effective duration varies depending on the injection site, dosage, and etiology of disease [19]. Currently, there is no high-quality evidence for the efficacy of BTX on neurogenic dysphagia [7, 8]. Thus, well-designed, randomized controlled clinical studies are needed to verify the efficacy and safety of BTX and to provide evidence for clinical applications.

The precise localization of the cricopharyngeal muscle during BTX injection is difficult to obtain and has been the focus of several studies. Since 1994, when Schneider [20] first described BTX injection into the cricopharyngeal muscle as an alternative to surgery, many researchers have tried different localization methods to ensure the efficacy of this treatment. Initially, BTX was injected under direct visualization during transcervical surgery and general anesthesia. Later, investigators attempted injecting BTX percutaneously, under the guidance of electromyography (EMG) or endoscope [16, 18, 21, 22]. Some researchers have injected BTX with the guidance of computed tomography (CT), which was shown to be effective and safe [23]. However, each guiding techniques has disadvantages if used alone. A new localization method will be used in this study, which is guided using ultrasound, catheter balloon, and EMG.

Here, we present a study protocol designed to explore the efficacy and safety of BTX injection guided by ultrasound, catheter balloon, and EMG for treatment of neurogenic cricopharyngeal achalasia.

## Methods and design

### Objective

To explore the efficacy and safety of BTX for treatment of neurogenic cricopharyngeal achalasia, with BTX injection guided by ultrasound, catheter balloon, and electromyography, a novel precise positioning method.

### Design

The administration of BTX (BOTOX, Allergan, Inc., Irvine, CA, USA) for cricopharyngeal dysfunction guided by a combination of catheter balloon, ultrasound, and electromyography in patients with stroke (BECURE) is a prospective, randomized controlled, double-blinded, superiority, single-center clinical trial. This study was approved by the Ethics Committee of the Third Affiliated Hospital of Sun Yat-sen University ([2019]02-419-01).

### Patient population

Patients with stroke and dysphagia who failed to improve after at least 1 week of conventional dysphagia rehabilitation treatment, including balloon dilation.

### Study setting

This study will be conducted in an academic hospital, the Third Affiliated Hospital, Sun Yat-sen University in Guangdong Province, China.

### Inclusion criteria

Patients are eligible for study participation if they fulfill all of the following criteria:

- Meet the diagnostic criteria for stroke, listed in the *Diagnostic criteria of cerebrovascular disease in China (version 2019)* [24].
- Stable vital signs without severe cardiopulmonary disease
- Aged 18–90 years
- Failed in swallowing function improvement after 1 week of balloon dilation
- Functional Oral Intake Scale (FOIS) Score ≤ 2
- Cricopharyngeal muscle cannot completely open, verified by video fluoroscopic swallowing study (VFSS); moderate-severe residue in epiglottic valley or piriform sinus, evaluated by flexible endoscopic evaluation of swallowing (FEES).
- UES residual pressure > 20 mmHg by high-resolution manometry (HRM).
- Cooperation with treatment without dysfunction of hearing and comprehension.
- Provide informed consent on their own behalf (or by legally appointed representatives/authorities).

### Exclusion criteria

Patients will be excluded from study participation if any of the following apply:

- Suffer from other neurogenic dysphagia or a condition that can cause dysphagia (for example, Parkinson’s disease).
- Have a non-neurogenic disease (e.g., cancer).
- Have a neuromuscular disorder (e.g., myasthenia gravis or motor neuron disease).
- Have psychopathy.
- Unable to cooperate with examination and treatment.
- Have infections or cuts at injection site.
- Allergic to botulinum toxin.
- Have coagulation dysfunction.
- Receive or have received anesthetics or any medicine that affects neuromuscular junctions within 1 month prior to participation.
- Participating in another study that could influence the results of BTX injection on the cricopharyngeal muscle.

### Drop out criteria

- Participants are found to be unable to complete the follow-up on time, or there is an unpredictable condition such as severe adverse event.
- Participants require not to participate in this research.

### Definitions

Early group: patients receiving botulinum toxin type A (BOTOX) injection immediately after randomization.

Late group: patients receiving placebo injection immediately after randomization and receive BOTOX injection 2 weeks after randomization if the FOIS score < 3.

Retreat group: patients within the “early group” with a score < 3 of FOIS 2 weeks after BOTOX injection and will receive another BOTOX injection.

**Fig. 1:**
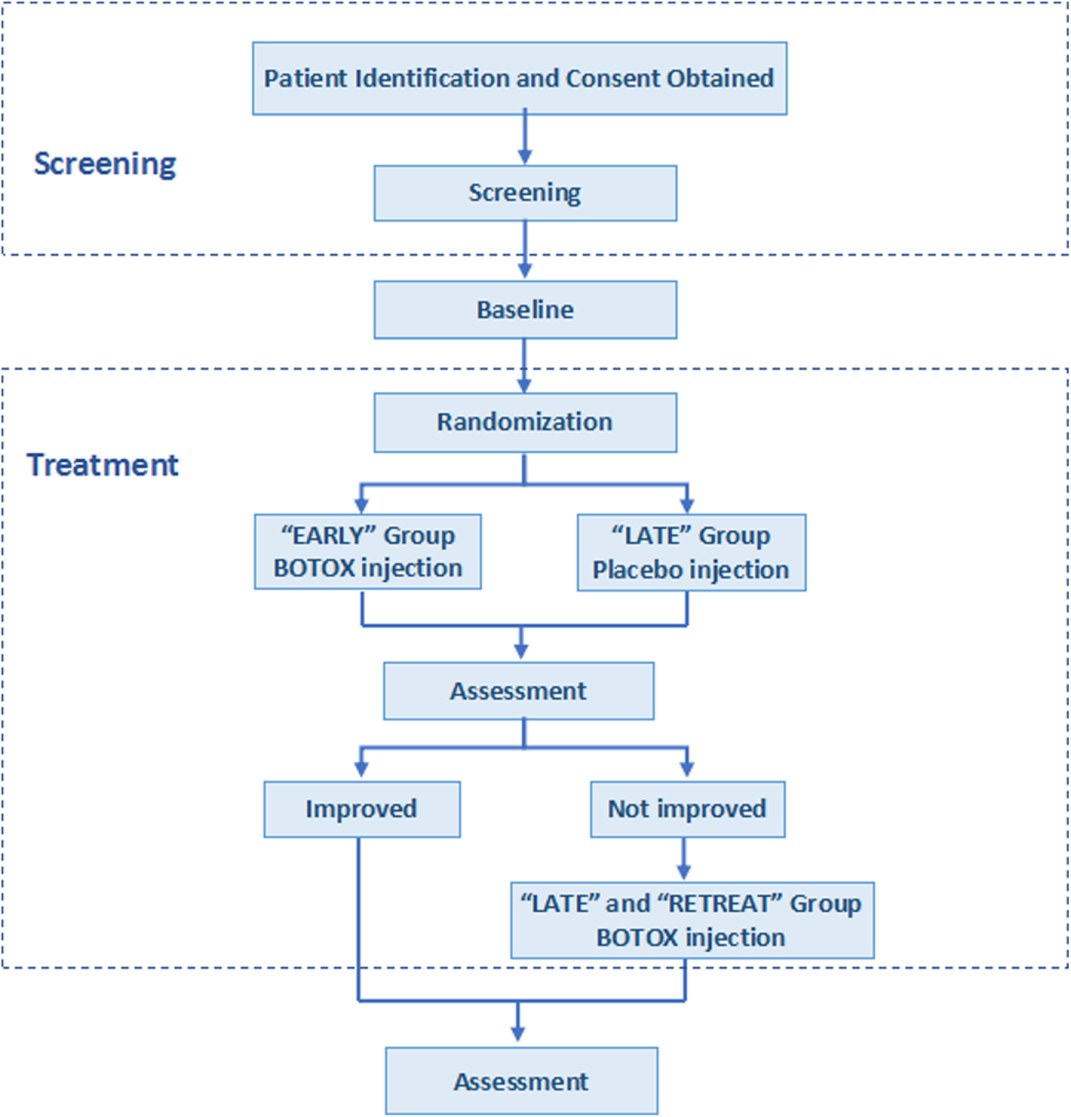
Graphical representation of the study design

Figure 1 is a graphical representation of the study design.

Explanation for choice of comparators:

The comparator will be normal saline (NaCl 0.9%, 0.5 ml). NaCl is the solvent of BOTOX, and therefore is considered a placebo.

### Allocation and randomization

The late group will initially act as the control group for the early group but will receive BTX treatment later if no improvement of FOIS is achieved. Qualified subjects will be randomized (1:1) in a double-blind fashion to the early group or late group (placebo-control). The randomization sequence was generated by a statistician using the Statistical Package for Social Sciences. A number from 1 to 44 was randomly generated and the random envelopes were generated according to the randomization sequence. The principle investigator (PI) is enrolling patients. A nurse who does not join in the assessment and treatment of the participants will assign participants to their allocation according to the random envelopes. Throughout the double-blind phase of the study, patients and investigators will not be informed of the treatment-group assignment. The BOTOX and placebo will be provided in identical syringes, allowing double-blinded administration. One nurse will be responsible for the dilution of BOTOX and placebo. Unblinding is permissible if there are any adverse events, or participants request the termination of the trial. When unblinding becomes necessary, the principal investigator (PI) will report the reason for the unblinding of patients and the associated adverse event (if exist) to the Data Monitoring Committee, who will jointly decide whether to unblind or not. Once unblinded, the participating investigators, the patient and his/her family will be informed of the patient’s allocation by the nurse who know the patient allocation. All interventions, examination results, adverse events, and reasons for unblinding will be documented.

### Treatment or intervention

The treatment will comprise of administering a BOTOX injection into the cricopharyngeal muscle guided by a combination of catheter balloon, ultrasound, and EMG. The BOTOX will be diluted to 100 U/ml with normal saline. Patients will receive 50 U (diluted in 0.5 ml normal saline) of BOTOX or a placebo (0.5 ml normal saline) in the cricopharyngeal muscle. First, the catheter balloon will be inserted into the esophagus through the nasal cavity, and 4 to 6 ml of water will be injected into the balloon. The balloon will be fixed at the cricopharyngeal muscle when pulled up softly and cannot be pulled up more. One person (speech language pathologist) will hold the catheter and maintain the balloon at this position. Second, ultrasound probe will be placed on the left or right side of the anterior cervical region. Under the guidance of ultrasound, the sternocleidomastoid muscle, thyroid gland, cricopharyngeal muscle, and balloon will be visible. The balloon, as a reference, will be fixed at the distal part of the cricopharyngeal muscle. The concentric EMG needle will be inserted in the marked side under the guidance of ultrasound. When the needle passes through the tissue and reaches the cricopharyngeal muscle, the EMG signal of this muscle can be heard, and then the patient will be instructed to swallow, allowing changes in the EMG signal to be observed and heard. After confirming the position, BOTOX will be injected into the target muscle. Generally, a single injection of botulinum toxin is 50 U. The BTX intervention will be applied by the PI (WHM) assisted by other investigators. Participants will remain hospitalized for the duration of the research. Every concomitant care and conventional dysphagia rehabilitation treatment will be allowed. Novel rehabilitation treatment such as repetitive transcranial magnetic stimulation is prohibited during the trail.

Every effort will be made to improve patient adherence to protocols. In the case of an adverse event following treatment administration (hoarseness, gastroesophageal reflux, local bleeding, aspiration pneumonia), treatment will be immediately discontinued.

### Primary outcomes

The Functional Oral Intake Scale (FOIS) [25] will be the main observational indicator used for the overall evaluation of swallowing function.

### Secondary outcomes

- UES residual pressure, evaluated by high-resolution manometry (HRM).
- UES resting pressure, defined as mean UES pressure calculated as the average of the inspiratory and expiratory values [26], with a reference range between 58 and 109 mmHg, evaluated by HRM.
- Duration of UES relaxation, evaluated by HRM.
- Velopharyngeal peak pressure and hypopharyngeal peak pressure, evaluated by HRM.
- UES opening, video fluoroscopic swallowing study (VFSS) (DBA-300 type remote control dual-bed gastrointestinal X-ray machine, Toshiba, Japan) will be used for evaluation, with the most open part of the cricopharyngeal muscle divided into 4 levels according to the modified barium swallow impairment profile (MBSImP) [27], corresponding to 0 to 3 points, respectively: normal open , 0; partially open, 1; slightly open, 2; and not open, 3.
- Pharyngeal construction ratio (PCR): evaluated by VFSS. It is the ratio of pharyngeal area at maximal contraction to pharyngeal area at rest [2]. The calculation formula: PCR = PA_max_/PA_hold_ (PA_max_ refers to the minimum area of the pharyngeal cavity when contracting during oral swallowing in the image of the VFSS, and PA_hold_ refers to the maximum area of the pharyngeal cavity with a 1 ml bolus in the patient’s mouth in the resting state). The calculation formula: PCR = PA_max_/PA_hold_ (PA_max_ refers to the pharyngeal area at maximum construction; PA_hold_ refers to the pharyngeal area with the bolus in the “hold” position) [28].
- Residue of bolus in the epiglottis valley or piriform sinus, evaluated by flexible endoscopic evaluation of swallowing (FEES).
- Penetration and aspiration, evaluated by VFSS and FEES.

### Participant timeline

**Fig. 2:**
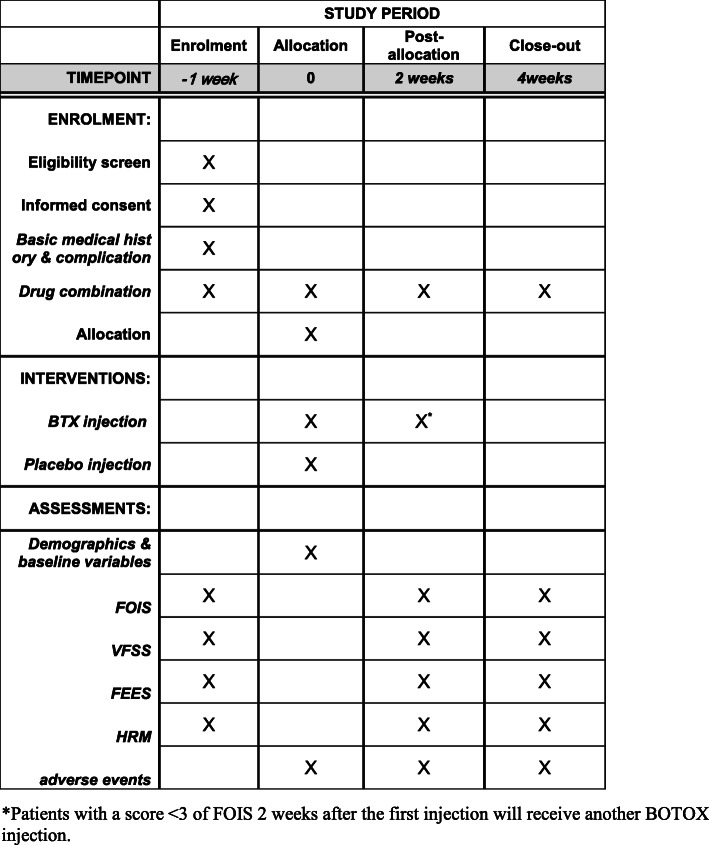
Schedule of enrolment, interventions, and assessments for all study patients

The total duration of participation in the study will be 4 weeks. Figure 2 is a schedule of enrollment, interventions, and assessments for all study patients.

### Sample size estimates

To detect significant differences between groups using an independent sample *t* test, a sample size of 44 patients is needed, assuming a spontaneous recovery mean ± SD in the late group of 2.6 ± 1.1 [29, 30], and a recovery mean ± SD of 4 ± 1.8 in the early group, as determined by our pilot study. These estimates assume a type I error of 0.05, a power of 0.80, and an estimated 10% rate of loss to follow-up.

### Recruitment

Patients with post-stroke dysphagia admitted to the Department of Rehabilitation Medicine, the Third Affiliated Hospital, Sun Yat-sen University, will be screened by an experienced doctor. In addition, the research information is posted on official accounts of our department. We also actively contact with other hospitals and ask them to refer potential suitable patients to our department. Those who meet the inclusion criteria and provide written informed consent will be enrolled.

### Data collection and management

A clinical research associate will ensure that patient inclusion and data collection are in line with the protocol and will check the following variables: patient initials, date of birth, sex, signed consent form, eligibility criteria, date of randomization, treatment assignment, adverse events, and study endpoints. The data will be collected by the clinical research associate on case report forms. The study documents will be stored and kept in a locked and secure office. All personnel involved in data analysis will be masked. Only the PI and the statisticians will have access to the final data set. If the patient drops out, all data before he/she drops out will be recorded, as well as the reason for the patient’s withdraw and any adverse event.

### Statistical analyses

The statistical analysis will be performed using the Statistical Package for Social Sciences (SPSS) version 23.0 for Windows by a statistician who is not involved in the study. A chi-square test will be used to compare the expulsion rate between the early and late groups. If the expulsion rate of the two groups is not statistically significant (*p* > 0.05), compliance of the groups will be considered similar. On the contrary, it is considered that the compliance of the two groups of patients is inconsistent, and it is necessary to re-recruit research cases, which will be assessed by the Data Monitoring Committee. Normally distributed data will be presented as the mean ± standard deviation. Categorical data will be presented as the frequency and proportions. Assumptions of normality will be tested with the Kolmogorov-Smirnov test. The parametric data will be analyzed by the independent sample *t* test, while the non-parametric data will be analyzed by the rank sum test. The *p* values ≤ 0.05 will be considered statistically significant. A comparison between two groups will be used to assess whether the efficacy (represented by FOIS, UES residual pressure, UES resting pressure, duration of UES relaxation, etc.) is different. A chi-square test will be used to compare the differences in the proportion of patients with improved swallowing function (assessed by FOIS) between the groups. Improvement prior to and post treatment in both groups will be assessed. Efficacy evaluation indicators (FOIS, UES residual pressure, UES resting pressure, duration of UES relaxation, etc.) will be compared two weeks after the first and second injections to assess improvement in the groups. A logistic regression method will be used to establish a probability model of BTX efficacy and influencing factors. The effect of each influencing factor on BTX efficacy (OR and 95% confidence interval) will be quantitatively analyzed to determine the interpretable accuracy and reliability of the coefficient *R*^2^ evaluation of the established efficacy and influencing factors model, with the FOIS score of the BTX group will be defined into effective and non-effective, a two-category variable *Y* (FOIS ≥ 3, *Y* = 1; FOIS<3, *Y* = 0). Patients’ age, gender, stroke lesion location, UES residual pressure, UES resting pressure, duration of UES relaxation, velopharyngeal peak pressure and hypopharyngeal peak pressure, UES opening, pharyngeal construction ratio, residue of bolus in the epiglottis valley or piriform sinus, penetration, and aspiration will be used as influencing factors of variable Xi (*i* = 1, 2, …11). A chi-square test will be used to compare the incidence of adverse events between the groups. An interim analysis will be performed after inclusion of the first 22 patients by a statistician who is not involved in this research. The data collection of the study will be assessed and confirmed by the Data Monitoring Committee, which can make decision to stop the research when it is harmful or futile.

### Adverse events

Adverse events and unintended effects during the treatment will be reported directly to the sponsor. The investigator will also assess the severity, causality, and seriousness of the adverse events and will inform the Data Monitoring Committee within 3 days of the initial observation of the event. The onset date, manifestations, severity, duration, action taken, and outcome for each adverse event will be recorded. Patients who suffer harm from trial will be cared for and financial compensation will be provided by our department.

### Monitoring

The principle investigator, who will be responsible for the monitoring and management of the study, will hold monthly research meetings. The hospital will monitor and conduct random audits of the research. The Data Monitoring Committee, which is independent from sponsors, will monitor and conduct random audits of the research. The monitoring and auditing will be performed in accordance with the sponsor’s monitoring and audit policies and procedures. The principle investigator will report to the sponsor every year with the following information: delegation log, adverse event log, deviation log, and annual progress reports to the Ethics Committee. Important protocol modifications will be communicated to the Ethical Committee and the trial registries. Every protocol amendment will be submitted to the Ethical Committee and sponsor.

## Discussion

The injection of BTX into the cricopharyngeal muscle can be effective for neurogenic dysphagia [7, 16–18]. However, no well-established evidence exists for its efficacy in treating neurogenic dysphagia. The efficacy varies depending on the injection site, dosage, and type of disease, among which, precise injecting into the cricopharyngeal muscle is the most difficult part. Common guidance methods such as EMG guidance, endoscopic guidance, and CT guidance have different advantages and disadvantages. We will take advantage of the experience of catheter balloon dilation in CPD [15], a newly developed application of catheter balloon, that is, a novel precise locating method using a combination of ultrasound, catheter balloon, and EMG in the guidance of BTX injection into the cricopharyngeal muscle. Under ultrasound guidance, a water-filled balloon will be fixed in the cricopharyngeal muscle, through which the thin cricopharyngeal muscle can be visualized easily. The EMG needle can confirm the muscle location by spontaneously measuring electroactivity, and the needle insertion is safe, as it is performed under ultrasound guidance. Our pilot study demonstrated the efficacy and safety of this novel technique. This randomized controlled study is designed to extend the results of existing research and provide stronger evidence for clinical application. This study has some limitations. This is a single-center study. The follow-up period of this study is relatively short.

## Trial status

The protocol version is 2.0, 20 May 2019. Participant recruiting started in September 2019. At the time of manuscript submission, we have included 10 participants in the study and they have completed the intervention. The completion of recruitment is anticipated to be at 1 June 2021. The completion of the final follow-up is anticipated to be at 30 June 2021.

## Data Availability

Only the PI and the statisticians will have access to the final data set. The data sets of this study will be available from the corresponding author on reasonable request, after publication.

## Abbreviations

BTX: Botulinum toxin
CPD: Cricopharyngeal dysfunction
CT: Computed tomography
EMG: Electromyography
FEES: Flexible endoscopic evaluation of swallowing
FOIS: Functional Oral Intake Scale
HRM: High-resolution manometry
MBSImP: The modified barium swallow impairment profile
PCR: Pharyngeal construction ratio
PI: Principal investigator
UES: Upper esophageal sphincter
VFSS: Videofluoroscopic swallowing study
